# Dentists’ attitudes towards chairside medical conditions screening in a dental setting in Saudi Arabia: an exploratory cross-sectional Study

**DOI:** 10.1186/s12903-019-0870-x

**Published:** 2019-08-06

**Authors:** Saba Kassim, Badr Othman, Sakher AlQahtani, Alemad Mustafa Kawthar, Sterling M. McPherson, Barbara L. Greenberg

**Affiliations:** 1 Department of Preventive Dental Sciences, Taibah University Dental College & Hospital, Al-Madinah Al-Munawwarah, 42353 Saudi Arabia; 2 Department of Pediatric Dentistry and Orthodontics, College of Dentistry, King Saud University, Riyadh, 11545 Saudi Arabia; 3 Pediatric Division AlJouf Specialty Dental Centre, Ministry of Health, AlJouf, Saudi Arabia; 4 Elson S. Floyd College of Medicine, Washington State University, Spokane, WA 9921-1495 USA; 5 Touro College of Dental Medicine, New York Medical College, Valhalla, NY USA

**Keywords:** Dentists, Saudi Arabia Oral health, Medical screening, Attitudes

## Abstract

**Background:**

Screening for medical conditions (MCs) of public health importance is a first step in disease prevention and control. Prior studies in the United States found oral health care providers (OHCPS) embrace screening for increased risk of medical conditions in the dental setting. Our objectives were to assess Saudi Arabian (SA) dentist’s attitudes, willingness and perceived barriers towards implementing screening for MCs into their dental practices.

**Methods:**

A self-administered, 5-point Likert Scale (1 = very important/willing to 5 = very unimportant/unwilling) questionnaire was given to a convenience sample of 190 practicing dentists. Friedman nonparametric analysis of variance was used to compare responses within each question.

**Results:**

Of the 143 responding dentists the mean age was 31 years; 102 (71%) were men. The majority felt it was important for a dentist to screen for cardiovascular disease (98.6%), hypertension (97.9%), diabetes (97.9%), human immunodeficiency virus (HIV) (97.9%), and hepatitis C virus (98.6%). Respondents were willing to refer a patient to a physician (97.9%); send samples to an outside laboratory (96.1%); conduct screening that yields immediate results (96.2%); and discuss results immediately with the patient (93.7%). Respondents were willing to measure/collect blood pressure (67.2%); weight and height (63.7%); and finger stick blood (54.6%). The whole responding dentists (100%) reported time as an important barrier. Respondents were significantly more willing to refer a patient for consultation than send samples to an outside laboratory (mean ranks: 2.32, 2.81, *P* < 0.001); significantly more willing to measure blood pressure than take oral fluids for salivary diagnostics (mean ranks 2.22, 2.75, *p* = 0.003). Insurance was significantly (*P* < 0.05) less important barrier than time, cost, patients’ willingness or liability (mean ranks 3.56, 2.63, 3.00, 2.79, 3.02, respectively).

**Conclusions:**

The majority of dentists in this study reported positive attitudes towards and willingness to perform medical screenings in their practice. Time was an important factor.

## Background

The current World Health Organisation (WHO) report estimates that 16 million people die prematurely aged 70-years-old from non-communicable diseases (NCDs) e.g. cardiovascular diseases [CVD] and diabetes [DM]); these are significant causes of death in developed and developing countries [1]. This is considered as a public health concern [2] and one of the major developmental challenges of the twenty-first century [1]. A set of ten global progress monitoring indicators towards achieving the 2030 Agenda for sustainable development was proposed [1]. Saudi Arabia (SA) is one of the member states that signed on to monitor the set of ten progress indicators that include implementing a multi-sectoral national strategy/action plan that addresses the major NCDs and their shared risk factors (e.g. tobacco use) [1, 3, 4]. The current data for SA is alarming, the percentage of deaths from NCDs is 78%, the total number of NCDs deaths is 70,000 and the probability of premature mortality from NCDs is 17% [1].

Successful disease prevention and control are most likely to require an integrated approach across multiple disciplines [5, 6]. Oral health is integral to overall health [7], notably, the bidirectional association of periodontal diseases and diabetes has been currently established [8]. Thus, dental visits represent an opportunity to provide screening to identify patients at or increased risk for a range of medical conditions (MCs) that include non-communicable (NCDs) e.g. diabetes, cardiovascular and infectious diseases e.g. human immunodeficiency virus infection (HIV) and Hepatitis C [5, 9–11]. Whilst this approach may target patients who do not see a physician, it is an additional heath care site to identify those who are unaware of their disease and who experience an increase in disease severity [9, 10, 12]. Notably, chairside medical condition screenings in a dental setting could provide a portal entry into primary care system as well as enhancing overall health outcomes [9, 13].

NCDs like diabetes and CVD are often underdiagnosed. For example, in the US an estimated 7.6 million, or 3.1% of American adults have undiagnosed diabetes. Additionally, about 81.6 million, or 33.9%, of American adults have prediabetes [14]. The average lag between onset and diagnosis is 7 years [15]. In SA, the available literature reported, from major city Jeddah at the Ambulatory Care Centres, King Abdulaziz Medical City, that of 507 participants (aged 20–40 years both genders) undiagnosed with cardiovascular risk factor of high blood pressure, 140 mmHg and/or diastolic blood pressure ≥ 90 mmHg, accounted for 8.3 and 0.6% had random blood glucose of ≥200 mg/dL [16].

There are available chairside screening tests for diabetes that include haemoglobin A1C and gingival cervicular blood (GCB) [8]. Screening tests for CVD include the Framingham risk score and heart score [17–19]. These tests that require a blood sample for testing have been assessed using safe, well validated and effective screening tools in dental settings [9, 10]. As for HIV rapid oral fluid tests could be administered at the start of a routine visit, with results available within 20 min [11].

Recent studies assessing dentists’ attitudes, willingness and barriers to give chairside screening for medical conditions reported that dentists felt screening for medical conditions was important and were willing to incorporate screening into their practice. However, barriers were also reported such as patients’ willingness and time constraints [5, 20]. In SA dentists utilize the routine guidelines as set by the Commission of Health Specialities that include medical history, dental history, extra and intra oral examination, radiographically examination, diagnosis, treatment planning, treatment and maintenance. Studies that assess SA dentists’ attitudes and willingness alongside barriers that hinder implementation screening for medical conditions in dental practice have not yet been done.

In light of the aforementioned research and the current report of the World Health Organization [1], it is timely to assess SA dentists’ attitudes towards screening for increased risk of MCs and related risk. Specifically, this is to meet the SA national NCDs’ goals for 2030 [1] as well as emphasising the World Dental Federation (FDI) to keep oral health on NCDs’ agenda [21]. This could also enable a more complete understanding of barriers and facilitators to implementing preventive health strategies that are conducive to both oral and general health and to subsequently alleviate the burden of NCDs including other MCs (e.g. HIV) that require minimum resources and efforts. Importantly, the findings of this study may influence the guidelines of the Saudi Commission of Health Specialties with respect to implementing relevant preventive measures in dental practices. Therefore, an exploratory small survey is valuable to gain an initial sense of SA dentists attitudes towards MCs for conducting a larger study [22]. The aim of this exploratory study was to assess practicing dentists’ attitudes, willingness and perceived barriers towards implementing screening for medical conditions into their practices in SA.

## Methods

### Participants sampling and setting of the study

This was a paper-based survey among a convenience sample of 190 (general dentists and specialists) dentists in Riyadh. Riyadh, the capital of SA is the most populated city in SA with a population of nearly five million, of which 69% are Saudis [23]. Dentists recruited into the study, between August and December 2017, included a consecutive sample of eligible (licensed) practicing dentists, regardless of their nationality (Saudi, non-Saudi), age, gender, locality of practicing in SA and specialty. All the dentists were offered to participate in the study during their attendance of the monthly meeting of the Saudi Dental Society (SDS). The latter was established in 1981 with many aims including the promotion and diffusion of interest in dentistry and dental research [24]. Given that this study was designed as exploratory, there was no need for sample size and power calculations.

### Measurement

A self-administered questionnaire consisting of pre-tested validated questions was used to collect the data (2) (available on request from last author). The questionnaire composed of two sections: section one asked about sociodemographic (e.g. age, gender) characteristics and section two about attitudes, willingness and perceived barriers of dentists towards chairside screening for increased risk of select medical conditions in the dental setting. Medical conditions selected included cardiovascular disease, diabetes, HIV, and hepatitis C. Barriers noted included time, cost, patient willingness, liability and medical insurance. Responses for section two were on a five-point Likert Scale (1 = very important/very willing; 5 = very unimportant/very unwilling). The questionnaire took between 5 and 8 min to complete. Formal permission was obtained from the SDS to collect the data.

### Statistical analysis

Data analysis was performed using the Statistical Package for Social Sciences Software (SPSS) for windows version 24 (IBM Corp, Armonk, New York, USA). Only four demographic variables contained missing values. Notably, ‘Year of graduation’ had 48% missing data, which was the highest amount. The lowest percentage of missing data was 28% in ‘Years Practicing’. While this one variable had a high amount of missing data, given the small number of variables with missing data we still opted to utilize multiple imputation and made use of non-parametric statistics in order to reduce bias as much as possible in our analyses. Missing data was handled using multiple imputation, which generated 70 datasets to maximize statistical efficiency. The statistical analysis plan was based on previous relevant studies [5, 25, 26]. Descriptive statistics (mean ± SD and frequency and percentages) was performed to report sample sociodemographic characteristics and attitudes and willingness of dentists towards chairside screening for medical conditions in their setting. The Friedman 2-way nonparametric analysis of variance (ANOVA) was conducted to calculate the mean rank sum value (lower mean rank score indicates very important and important/ very willing and willing) and to test whether there was a significant difference in the distribution of responses for each of the related items in a given question. If the Friedman test was significant at *p* ≤ 0.05, a post hoc pairwise comparison was run to identify significantly different pairs. We used the Bonferroni correction method for multiple comparisons as for the parametric ANOVA analyses and reported the adjusted significant *p*-value. The significance level was set at p ≤ 0. 05.

## Results

### Sample characteristics

Of the 190 distributed questionnaires 143 were returned giving a response rate of 75%. Table 1 shows the overall socio-demographics of the study sample. Of the responding dentists 102 (71%) were men and 90 (63%) were Saudi national.

**Table 1:** Sociodemographic characteristics of respondents (*n* = 143)

Variable	Frequency (%), or Mean ± SD
Age	30.96 ± 5.28
Nationality	
Saudi	90 (62.9)
Non Saudi	53 (37.1)
Specialty	
General practitioner dentist	64 (44.8)
Prosthodontist	20 (14.0)
Endodontist	14 (9.8)
Oral and Maxillofacial	10 (7.0)
Pediatric dentistry	14 (9.8)
Periodontist	9 (6.3)
Other (e.g. dental public health)	12 (8.4)
In practice	
≤10 years	101 (70.9)
>10 years	42 (29.1)
Locality	
Urban	130 (91.2)
Suburban	13 (8.8)

### Attitudes toward chairside screening for MCs

#### Attitude of responding dentists to identify patient with MCs

**Fig. 1:**
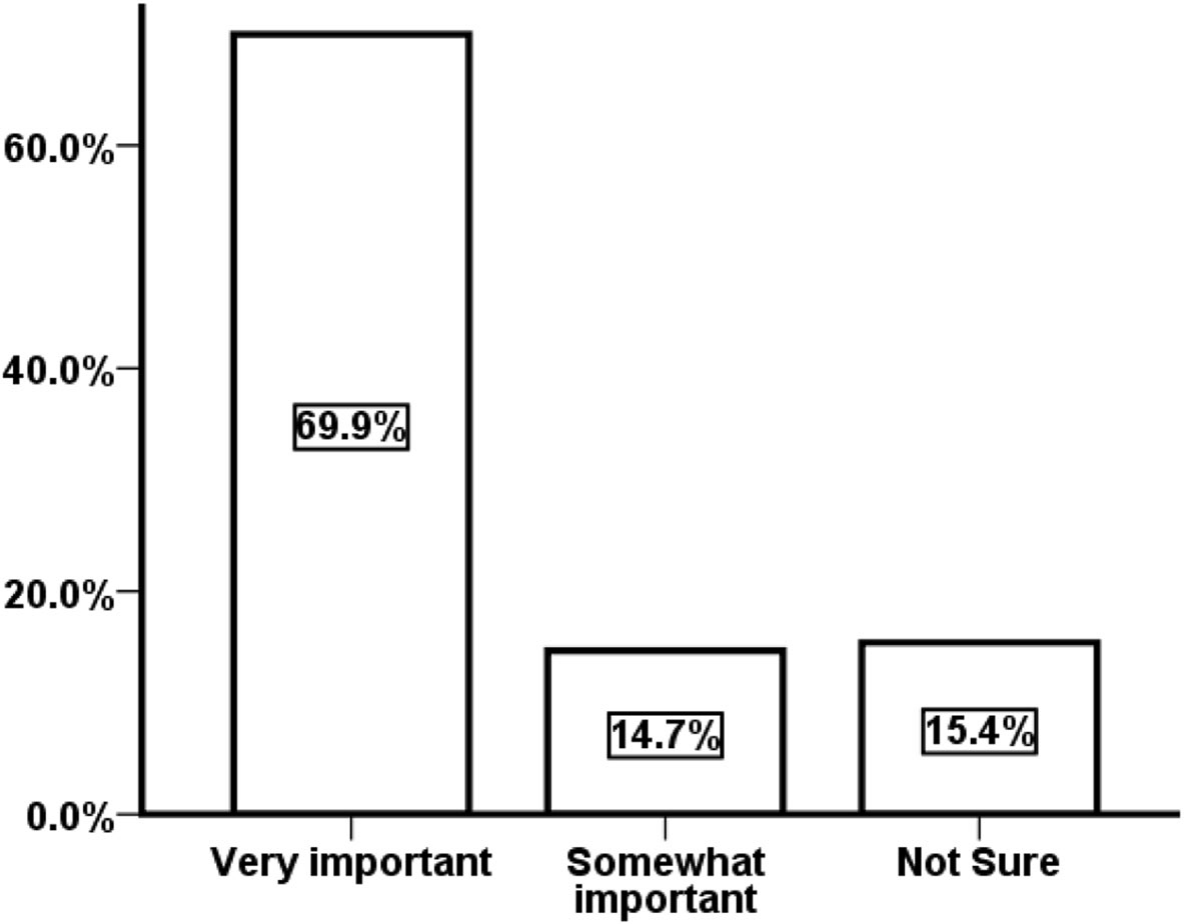
Respondents’ attitudes towards the important of identifying patients who may benefit from interventions to prevent or control the onset of MCs, (*n* = 143)

As for dentists’ attitudes towards the importance of identifying patients who may benefit from interventions to prevent or control the onset of MCs, approximately 85% or responding dentists thought that it was ‘Very important’ and ‘Somewhat important’ (Fig. 1).

#### Attitude of responding dentists to conducting chairside MCs screening

As shown in Table 2 most of the dentists in this sample responded with ‘Very important’ to Q1–4 about performance/conduct chairside screening for MCs. For example, 81.1% reported ‘Very important’ to screen for cardiovascular conditions and 79% for diabetes. Table 2 also shows the mean rank for every condition. A Friedman test was significant (χ^2^ [4] = 14.59, *p* < 0.006). As noted above and consistent with conventional ANOVA post hoc tests, we conducted pairwise comparisons. However, the observed differences were non-significant after adjustment, i.e. p = > 0. 05.

**Table 2:** Distribution of responses (frequency and percentages), mean rank and pairwise comparisons for Question 1–4, (*n* = 143)

1.1.How important do you think it is for a dentist to perform/conduct chairside screening for each of the following?	Very important (1) N (%)	Somewhat important (2) N (%)	Not sure (3) N (%)	Somewhat unimportant (4) N (%)	Very Unimportant (5) N (%)	Total	Median (IQR)^†^	Mean + rank	Pairwise comparisons					
										a	b	C	d	e
(a) Cardiovascular diseases	116 (81.1)	25(17.5)	1 (0.7)	1 (0.7)	0 (0.0)	143	1(0)	2.92	a					
(b) Hypertension	97 (67.8)	43 (30.1)	3 (2.1)	0 (0.0)	0 (0.0)	143	1(1)	3.24	b					
(c) Diabetes mellitus	113 (79)	27(18.9)	3 (2.1)	0 (0.0)	0 (0.0)	143	1(0)	2.98	c					
(d) HIV	112 (78.3)	28 (19.6)	2 (1.4)	0 (0.0)	1 (0.7)	143	1(0)	3.00	d					
(e) Hepatitis	121 (84.6)	20 (14.0)	1 (0.7)	0 (0.0)	1 (0.7)	143	1(0)	2.85	e					
Friedman test significant difference between the mean ranks *χ*^2^ (4) = 14.59, p < 0.006; *significantly different at *P* < 0.05														
2.If you were considering incorporating medical screening into your practice, how willing would you be to do each of the following?	Very willing (1) N (%)	Somewhat willing (2) N (%)	Not sure (3) N (%)	Somewhat unwilling (4) N (%)	Very unwilling (5) N (%)	Total	Median (IQR))^†^	Mean + rank	Pairwise comparisons					
										a	b	c	d	
(a) Conduct chairside screening that yields immediate results	89 (62.2)	43 (30.1)	9 (6.3)	1 (0.7)	1 (0.7)	143	1(1)	2.50	a					
(b) Conduct chairside screening that requires sending samples to an outside laboratory	68 (47.6)	55 (38.5)	16 (11.2)	3 (2.1)	1 (0.7)	143	2(1)	2.81	b				*	
(c) Discuss screening results with patients immediately following screening	90 (62.9)	44 (30.8)	7 (4.9)	2 (1.4)	0 (0.0)	143	1(1)	2.45	c					
(d) Refer a patient for consultation with a physician	106 (74.1)	34 (23.8)	1 (0.7)	1 (0.7)	1 (0.7)	143	1(1)	2.23	d		*			
Friedman test significant difference between the mean ranks *χ*^2^ (3) = 27.73, p < 0.001; *significantly different at P < 0.05														
3. How willing would you be to gather the following samples or data as part of your practice?	Very willing (1) N (%)	Somewhat willing (2) N (%)	Not sure (3) N (%)	Somewhat unwilling (4) N (%)	Very unwilling (5) N (%)	Total	Median (IQR))^†^	Mean + rank	Pairwise comparisons					
										a	b	c	d	
(a) Oral fluids for salivary diagnostics	33 (23.1)	34 (23.8)	49 (34.3)	19 (13.3)	8 (5.6)	143	3(1)	2.75	a			*		
(b) Drop of blood by finger stick	45 (31.5)	33 (23.1)	36 (25.2)	22 (15.4)	7 (4.9)	143	2(2)	2.58	b					
(c) Blood pressure measurements	56 (39.2)	40 (28.0)	26 (18.2)	14 (9.8)	7 (4.9)	143	2(2)	2.22	c	*				
(d) Height and weight (BMI)	49 (34.3)	42 (29.4)	24 (16.8)	14 (9.8)	14 (9.8)	143	2(2)	2.45	d					
Friedman test significant difference between the mean ranks *χ*^2^ (3) = 21.24, P < 0.001; *significantly different at P < 0.05														
4. If you were considering incorporating medical screening into your practice, how important would each of the following issues be?	Very important (1) N (%)	Somewhat important (2) N (%)	Not sure (3) N (%)	Somewhat unimportant (4) N (%)	Very Unimportant (5) N (%)	Total	Median (IQR)^†^	Mean + rank	Pairwise comparisons					
										a	b	c	d	e
(a) Time	110 (76.9)	33 (23.1)	0 (0.0)	0 (0.0)	0 (0.0)	143	1(0)	2.63	a			*		
(b) Cost	89 (62.2)	51 (35.7)	2 (1.4)	1 (0.7)	0 (0.0)	143	1(1)	3.00	b			*		
(c) Insurance coverage	66 (46.2)	48 (33.6)	20 (14.0)	7 (4.9)	2 (1.4)	143	2(1)	3.56	c	*	*		*	*
(d) Patient willingness	100 (69.9)	39 (27.3)	3 (2.1)	1 (0.7)	0 (0.0)	143	1(1)	2.79	d			*		
(e) Liability	90 (62.9)	44 (30.8)	7 (4.9)	2 (1.4)	0 (0.0)	143	1(1)	3.02	e			*		

Friedman test significant difference between the mean ranks *χ*^2^ (4) = 54.24, *p* < 0.001; *significantly different at *P* < 0.05

+ Lower mean rank indicates more important/ more willing; ^**†**^Median (IQR) = Median with interquartile range

#### Willingness to perform chairside screening

Approximately, all respondents were willing (97.9%) to refer a patient for consultation with a physician and this was followed by 96.1% to conduct screening that requires sending samples to an outside laboratory. Ninety-four (93.7%) respondents would be willing to discuss results immediately with the patient during the dental visit and do screening (92.3%) that yields immediate results (Table 2). A Friedman test was significant (*χ*^2^ [3] = 27.73, *p* < 0.001). Post hoc pairwise comparisons with adjusted *p*-value showed that only significant differences were in means ranks between “refer a patient for consultation with a physician” and “conduct chairside screening that requires sending samples to an outside laboratory” (mean ranks 2.23, 2.81, *p* = 0.001). Respondents were significantly more willing to incorporate chairside medical screening in their practice and refer a patient for consultation to a physician than conduct a medical screening that required sending samples to an outside laboratory.

#### Willingness to execute screening, gather samples or measurements

As for willingness to collect samples or measurements, Table 2 demonstrates that 67 and 64% dentists were ‘Very willing and Willing’ to take blood pressure and BMI Measurements. However, fewer dentists were ‘Very willing and Willing’ to gather ‘Oral fluids for salivary diagnostics’ and ‘Drop of blood by finger stick’, 48 and 55% respectively. The mean rank for most willing was for measuring blood pressure (2.22), followed by collecting BMI (2.45) and taking a drop of blood by finger stick (2.58) and the least willing was for collecting oral fluids for salivary diagnostics (2.78). An overall Friedman test was significant (*χ*^2^ [3] = 21.24, *p* < 0.001). The post hoc pairwise comparisons analysis with adjusted *p*-value (Table 2) showed that responding dentists were significantly (mean ranks 2.22, 2.75, *p* = 0.003) more willing to ‘Measure blood pressure’ than ‘Collect Oral fluids for salivary diagnostics’, and no differences were observed between other measurement/ data collection items.

#### Perceived barriers for incorporating chairside medical conditions screening into practice

Time was reported by the whole responding dentists (100%) as important for incorporating chairside MCs screening into practice, followed by ‘Patient willingness’ and ‘Liability’ (97,93% respectively). Insurance coverage was reported as less important (77%). A Friedman test was significant (χ^2^ [4] = 54.24, p < 0.001). Post hoc pairwise comparisons with adjusted p-value showed significant differences only in means ranks between insurance and all barriers; time and insurance (mean ranks 2.63, 3.56, *p* = 0.001), cost and insurance (mean ranks 3.00, 3.56, *p* = 0.031), patient willingness and insurance (mean ranks 2.79,3.56, *p* = 0.001) and liability and insurance (mean ranks 3.02, 3.56, *p* = 0.045). Insurance coverage was ranked as significantly less important as a perceived barrier than all other potential barriers noted.

## Discussion

To the best of our knowledge this is the first study that explores practicing dentists’ attitudes and willingness to screen patients for MCs in a specific region of the Middle East and North Africa (MENA) i.e. Saudi Arabia. Generally, the majority of the surveyed dentists had positive attitudes and were willing to conduct chairside screening in their dental setting. These findings aligned with other relevant studies that were conducted among dentists and dental hygienists over the last 10 years in the United States (US), United Kingdom (UK) and India [5, 8, 20, 25, 27].

In this survey, almost all of our responding dentists reported the importance to perform chairside screening for CVD, HIV and hepatitis and these findings were as well aligned with other studies reported in the US [5, 25]. With respect to willingness to perform chairside screening our results are in accordance with studies conducted among dentists and hygienists in the US [5, 25]. Specifically, willingness to refer patients to physicians was reported by almost all dentists and this was followed by approximately an equal percentage of dentists willing to conduct screening that yielded immediate results and discussion of results with patients. Interestingly, in our study almost all the respondents were positive about screening for HIV which is in contrast to what was reported among US dentists [5]. This could be attributed to that in the US where HIV testing is more likely to be acceptable and available with less stigma. Notably, Siegel et al. [28] reported barriers to offer patients HIV testing in dental setting was to avoid offending patients, viewing HIV testing as outside the scope of licensure, anticipating low patient acceptance of HIV testing, expecting inadequate reimbursement, and a potential negative impact on the practice. However, in SA HIV testing is not accessible and caries a huge stigma, making it less likely that people will be tested, and therefore there is a great need for testing. As such the rapid HIV test in dental clinic in SA would hugely be beneficial to both patients and dental staff.

However, time was the major barrier to screen patients for MCs as it was reported in the UK [20]. For samples requiring saliva, this reluctance could be (for example) attributed to the time needed for collecting the saliva (stimulated, unstimulated), the storage, the process of shipment of the saliva, in addition to extra time needed for infection control [29]. Having, insurance was the least important barrier to incorporating screening into the dental practice. Our findings were consistent with studies in the US [5].

There are both strengths and limitations to this study. Strengths: the use of validated questionnaire [5] allowed us to compare our study findings as well as the diversity of the sample with respect to recruited dentists from different specialties. Limitations: Our sample was small and not representative (convenience sample) of all practicing dentists in SA, as such the generalizability of the study results is limited. This was an exploratory study that will provide supporting data to conduct a nationally based study with a more representative study sample of dentists in SA. The suggested perceived barriers in the survey for may have not captured context-specific barriers. However, in order to have data comparable to other similar studies we used similar barriers as were used in those studies. The social desirability and self-selection into the study may have biased the results i.e. respondents could be the motivated dentists. This limitation could be overcome in future research with an appropriate sample.

The acceptance of patients to be screened and the creation of referral pathways between dentist and medical physician and relevant dental care providers (e.g. dental hygienists) should be investigated as reported in the US [2, 5, 25–27, 30]. Attitudes toward screening in a dental setting among primary care physicians and patients’ attitudes in this region should also be explored.

As for the practical implications of the study, the alarming MCs burdening this region solidify the necessity of putting action in place. This small survey assessment of dentists’ attitudes to screen for increased risk of MCs helps bridge the knowledge gap in research and pave the way for a national survey investigating dentists’ attitudes toward medical screening in the dental practice. Subsequently, this could lead to implementation studies and establishing additional training opportunities for SA dentist and possibly incorporation into the dental school curriculum. The role of modifiable (e.g. education) and none modifiable (gender) factors in dentists’ attitudes towards MCs screening should be investigated in future research.

## Conclusions

This study sample of relatively young dentists supported the importance of screening patients for increased risk of medical conditions and demonstrated positive attitudes and willingness towards chairside screening, with time as the main perceived barrier to implementing medical screening in the dental setting. As this was a small survey among a convenience sample, there is a need to conduct a larger national survey in SA to corroborate the findings of this study and set the foundation for conducting an implementation study.

## Abbreviations

(OHCPS): Oral health care providers
HIV: human immunodeficiency virus
MCs: Medical Conditions
SA: Saudi Arabia
UK: United Kingdom
US: United States
